# Inhibitory Effect of Crocin(s) on Lens α-Crystallin Glycation and Aggregation, Results in the Decrease of the Risk of Diabetic Cataract

**DOI:** 10.3390/molecules21020143

**Published:** 2016-01-26

**Authors:** Fereshteh Bahmani, Seyedeh Zahra Bathaie, Seyed Javid Aldavood, Arezou Ghahghaei

**Affiliations:** 1 Department of Clinical Biochemistry, Faculty of Medical Sciences, Tarbiat Modares University (TMU), Tehran P. O. Box 14115-111, Iran; bahmani@kaums.ac.ir; 2 Department of Clinical Sciences, Faculty of Veterinary Medicine, University of Tehran, Tehran P. O. Box 14185-746, Iran; sja@ut.ac.ir; 3 Department of Biology, Faculty of Science, University of Sistan and Baluchestan, Zahedan P. O. Box 98167, Iran; arezou@chem.usb.ac.ir

**Keywords:** crocin(s), structure-function, streptozotocin, cataract, glycation, oxidative stress

## Abstract

The current study investigates the inhibitory effect of crocin(s), also known as saffron apocarotenoids, on protein glycation and aggregation in diabetic rats, and α-crystallin glycation. Thus, crocin(s) were administered by intraperitoneal injection to normal and streptozotocin-induced diabetic rats. The cataract progression was recorded regularly every two weeks and was classified into four stages. After eight weeks, the animals were sacrificed and the parameters involved in the cataract formation were measured in the animal lenses. Some parameters were also determined in the serum and blood of the rats. In addition, the effect of crocin(s) on the structure and chaperone activity of α-crystallin in the presence of glucose was studied by different methods. Crocin(s) lowered serum glucose levels of diabetic rats and effectively maintained plasma total antioxidants, glutathione levels and catalase activity in the lens of the animals. In the *in vitro* study, crocin(s) inhibited α-crystallin glycation and aggregation. Advanced glycation end products fluorescence, hydrophobicity and protein cross-links were also decreased in the presence of crocin(s). In addition, the decreased chaperone activity of α-crystallin in the presence of glucose changed and became close to the native value by the addition of crocin(s) in the medium. Crocin(s) thus showed a powerful inhibitory effect on α-crystallin glycation and preserved the structure-function of this protein. Crocin(s) also showed the beneficial effects on prevention of diabetic cataract.

## 1. Introduction

Cloudiness of the natural lens is called a cataract. Cataracts remain a major cause of visual disability and blindness worldwide and their prevalence in diabetic patients is fivefold higher than among non-diabetics [1]. Important evidence suggests that several long-term pathological consequences of diabetes, including cataracts, are created by the accumulation of macromolecules that have been modified by non-enzymatic glycation (reaction of sugars with the amino groups of proteins) and oxidation. It is believed that non-enzymatic glycation and oxidative damage of lens proteins are the major factors responsible for the diabetic cataract formation, by altering the lens protein structure and stability and inducing protein cross-linking, aggregation, and insolubilization [2,3]. α-Crystallin is a major structural protein of the vertebrate eye lens. This water-soluble protein is a member of the small heat shock protein family and has been known as a molecular chaperone in the eye lens. It inhibits the aggregation and inactivation of several other lens proteins and enzymes and hence plays an important role in maintaining the transparency of the lens [4]. Due to the long half-life of this protein [5,6], it is susceptible to several post-translational modifications, such as glycation and oxidation during aging and diabetes. Non-enzymatic glycation and oxidation of lens proteins and reduction in the chaperone function of α-crystallin have been reported previously in diabetic conditions [7,8]. According to World Health Organization data, cataract is the first cause of blindness, accounting for 51% of cases around the world [9]. At present, the surgical removal of the opaque lens and its replacement with an artificial one is the only available treatment for cataract. Because surgery is not very satisfactory, and on the other hand, surgical treatment is not widely available in developing countries and may have complications [1], many researchers have investigated pharmaceutical agents to prevent this disease. In the past decade, scientists have tested the protective effect of natural products against cataract [10].

Saffron (*Iridaceae* family), one of the most expensive spices in the world, consists of the dried stigmas of *Crocus sativus* L., and has been consumed since antiquity as a home remedy to cure various diseases [11]. Chemical analysis of saffron has shown the presence of more than 150 components. The most functional components of saffron are certain apocarotenoids, collectively known as crocin(s) and monoterpene aldehydes (picrocrocin and safranal). Crocin(s) are saffron-colored compounds and due to their high glycosyl contents, are unique water-soluble carotenoids in nature [12,13]. Good quality saffron contains about 30% crocin(s). Further studies have indicated the powerful antioxidant activity of crocin(s), which is higher than *α*-tocopherol and prevents the peroxidation of lipid products and partly restores the activities of some antioxidant enzymes [14]. Therefore, due to their powerful antioxidant activity and hypoglycemic properties [15], crocin(s) may prevent cataract formation in diabetes and protect proteins, especially α-crystallin, against glycation and oxidative damage, and hence, preserves the activity of proteins. In the present study, and in continuation of our previous works investigating the various biological effects of saffron and its carotenoids [11,15,16,17,18,19,20,21], we studied the protective effect of crocin(s) on the *in vitro* non-enzymatic glycation and oxidative damage of calf eye α-crystallin, and lens opacification in streptozotocin-induced diabetic rats.

## 2. Results

### 2.1. In Vitro Studies

We studied the effect of crocin(s) on glycation of α-crystallin as a model protein of the lens in the absence and presence of glucose and crocin(s). Incubation of α-crystallin with glucose (500 mM) induced glycation of this protein and increased its non-tryptophan, advanced glycation end product (AGE) fluorescence emission.

**Figure 1:**
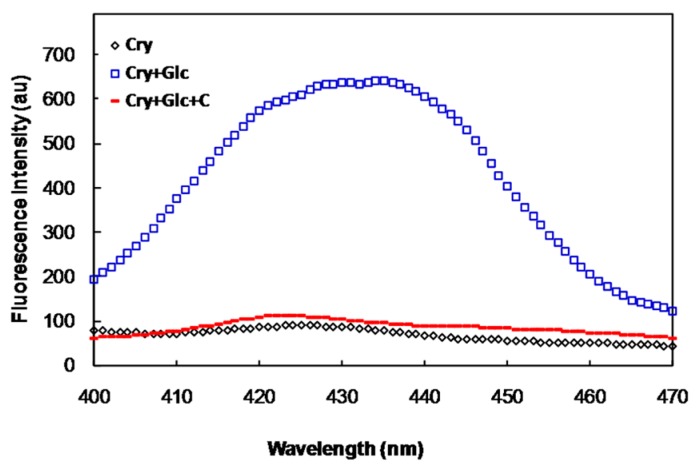
Non-tryptophan AGE fluorescence of α-crystallin (Cry), in the presence of 500 mM of glucose (Glc) with and without crocin(s) (C).

As can be seen in the Figure 1, the emission of α-crystallin AGEs fluorescence was lower in the presence of crocin(s). Crocin(s) alone had also no effect on the AGE fluorescence of this protein.

**Figure 2:**
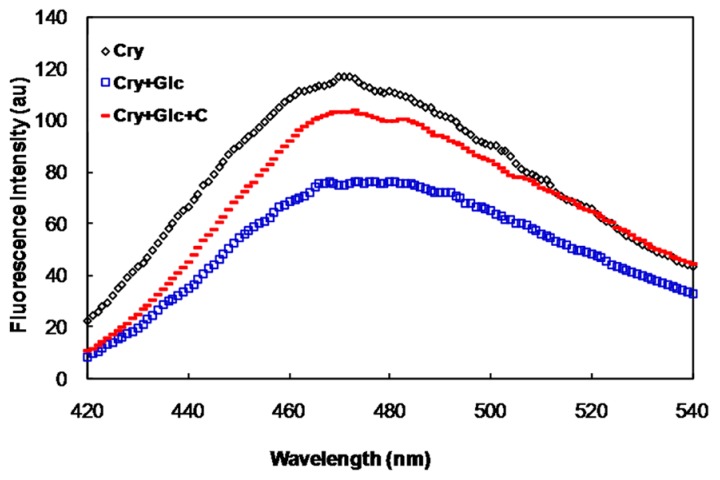
ANS fluorescence of α-crystallin, in the presence of 500 mM of glucose with and without crocin(s).

The surface-exposed hydrophobic residues of α-crystallin was investigated as a function of binding of 1-aniline 8-naphthalene sulfonate (ANS), hydrophobic probe, to this protein. The fluorescence intensity of glycated *α*-crystallin-ANS was lower than that of the native protein (Figure 2). However, this value was increased in the presence of crocin(s).

**Figure 3:**
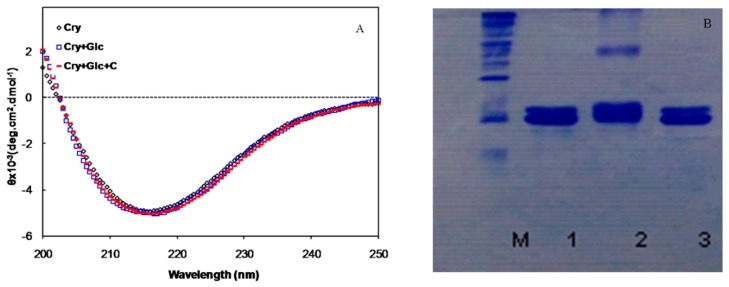
(**A**) Far-UV CD spectra of α-crystallin, in the presence of 500 mM of glucose with and without crocin(s); (**B**) Electrophoretic mobility of α-crystallin. M, Molecular Marker, 1-3, α-crystallin alone, in the presence of glucose and glucose+crocin(s), respectively.

The secondary structure of the protein solution was also investigated by circular dichroism (CD) in the far-UV region. There were no significant changes in the CD signal of the glycated *α*-crystallin in the presence and absence of crocin(s) (Figure 3A).

In addition, glycation of α-crystallin with glucose resulted in the cross-linking and formation of high molecular weight (HMW) aggregates that was searched by SDS-PAGE (Figure 3B). Some of the cross-linked protein also could not enter into the resolving gel and remains on top of the gel. Interestingly, the presence of crocin(s) in the medium prevented the formation of glycated protein cross-links and reduced HMW α-crystallin aggregates.

**Figure 4:**
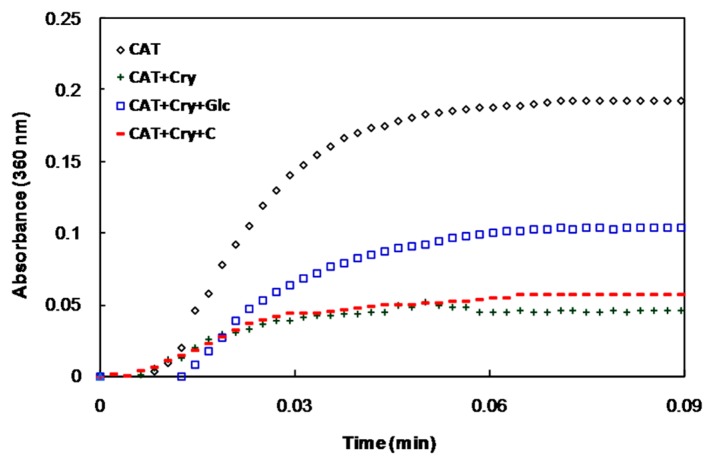
Chaperone activity of α-crystallin as measured by the suppression of heat-induced aggregation of catalase. Catalase was incubated at 60 °C in the absence and presence of native α-crystallin and in the presence of *α*-crystallin with glucose or glucose+crocin(s).

The chaperone activities of α-crystallin at different conditions were also investigated. The native form of α-crystallin protects catalase (CAT) against heat-induced aggregation. The ability of the glycated protein to protect CAT against aggregation was remarkably reduced, while the degree of protection of α-crystallin in the presence of crocin(s) (in the presence or absence of glucose) was similar to that of the native form (Figure 4).

### 2.2. In Vivo Studies

**Figure 5:**
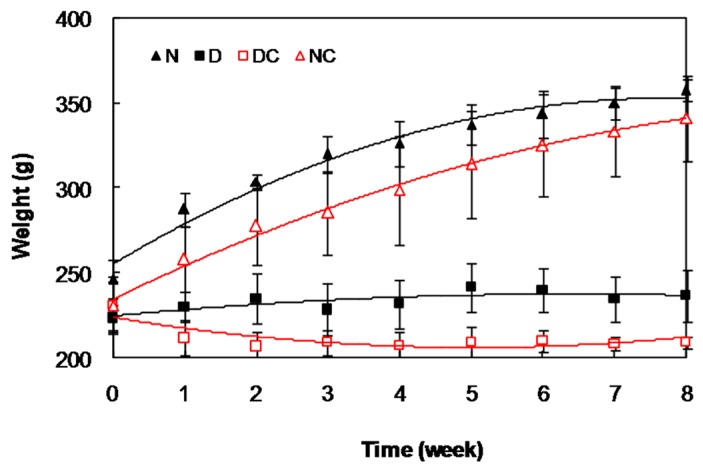
Changes in the body weight of all rat groups during the time course of the study.

The weight of all animals was determined weekly (Figure 5) and the results indicated a gradual decrease in the weight of diabetic group, so that after 8 weeks, it was significantly (*p <* 0.001) lower in the diabetic group than the control group (235.5 ± 16.9 g and 330.0 ± 16.7 g, respectively). The results indicated that crocin(s) had no significant effect on this parameter, in either diabetic or control groups.

**Figure 6:**
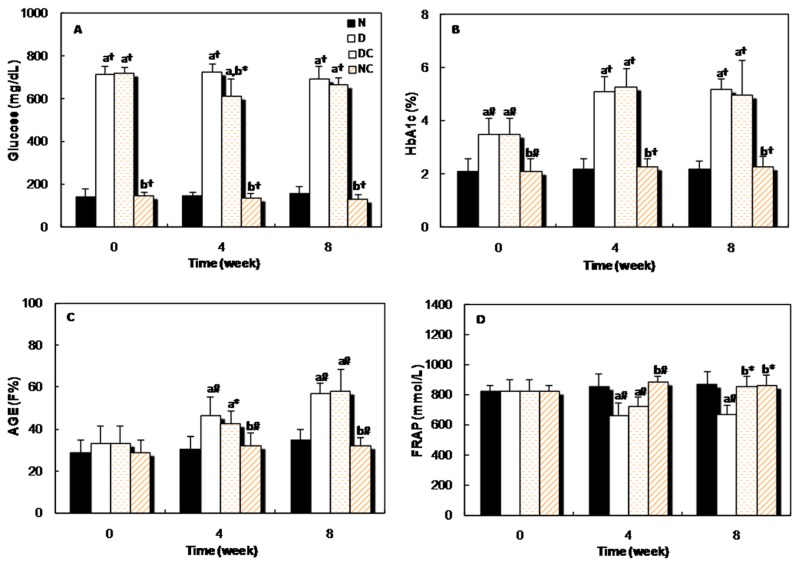
Changes in the (**A**): glucose concentration; (**B**): percent of HbA1c; (**C**): fluorescence intensity of AGEs; and (**D**): FRAP, in different groups of rats at the beginning (0 week), middle (4 weeks), and at the end (8 weeks) of the experiment. “a” indicates the significance of the data that compares group N *vs.* all groups. “b” indicates the significance of the data that compares group D *vs.* groups of crocin(s)-treated rats. *, *p <* 0.05; #, *p <* 0.01; and †, *p <* 0.001.

Figure 6A shows the changes in the serum glucose level of all four groups of rats at the beginning, middle and the end of the study. The glucose level in the sera of both diabetic groups was higher than the normal group, but four weeks after receiving crocin(s), the glucose concentration reduced significantly (*p <* 0.05) in the DC group. Figure 6B,C indicates that, the blood HbA1c and AGEs levels of diabetic rats increased gradually throughout the experimental period and significant differences (*p <* 0.001 and *p <* 0.01) were observed in these parameters between the diabetic and normal groups. Administration of crocin(s) had no significant effect on these parameters. The evaluation of the plasma antioxidant activity was performed by FRAP assay. This parameter decreased significantly in the diabetic group, but after 8 weeks of treatment with crocin(s), it increased significantly (*p <* 0.05, Figure 6D).

**Figure 7:**
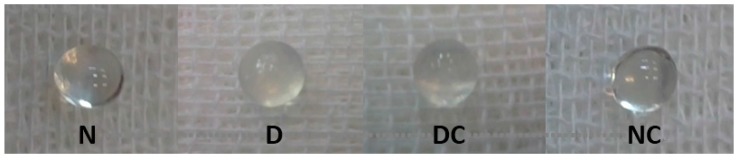
Change in the opacity of the lenses in different group of rats. N, Normal; D, diabetic; DC and NC, diabetic and normal rats received crocin(s).

Figure 7 shows the lenses of different groups of rats in this study. The opacification of the lens in the diabetic group was started five weeks after streptozotocin injection and diabetes induction (Table 1). Cataract was developed in all diabetic rats in a nearly similar rate. After eight weeks, 33.4% of the lenses of the D groups were in grade II, 66.6% in grade III and none of them were clear. However, in the diabetic group treated with crocin(s), 66.6% of the lenses were in grade II and 33.4% were in grade III. Crocin(s) had no cataractogenic effect on normal rats. These results indicated that crocin(s) treatment reduced the rate of cataract development in the diabetic group.

**Table 1:** The effect of crocin(s) on the average lens opacity in each group at different weeks.

Average of Cataract Score Time (week)					
Group	0	2	4	6	8
N	0	0	0	0	0
D	0	0	0.3	2	2.6
DC	0	0	0.3	1.5	2.3
NC	0	0	0	0	0

Cataract formation was scored biweekly according to the following classification: clear normal lens (0), peripheral vesicles (I), peripheral vesicles and cortical opacities (II), diffuse central opacities (III) and mature cataract (IV). The scores of cataracts in each group were averaged at the given time. The average lens opacity was lower in diabetic groups treated with crocin(s) at 6 and 8 weeks of the experiment.

*Biochemical Analysis of the Lens:* The amount of glutathione (GSH) and activity of CAT decreased in the lenses of diabetic rats (Table 2). Administration of crocin(s) had a statistically significant (*p <* 0.01 for GSH and *p <* 0.05 for CAT) effect on GSH and CAT, respectively and increased these antioxidant markers. Conversely, as Table 2 shows, hyperglycemia increases the activity of the superoxide dismutase (SOD) in the lenses of the diabetic group. No significant changes were observed in the SOD activity due to crocin(s) treatment.

**Table 2:** The effect of crocin(s) on the glutathione content and the activities of antioxidant enzymes in the rats lenses.

Group Name	GSH (mg/g Lens)	CAT Activity (μmol/g Lens pr)	SOD Activity (Units/min/mg Lens pr)
N	214.5 ± 43.2	57.6 ± 2.4	6.59 ± 0.95
D	54.9 ± 12.7 ^a,†^	31.1 ± 5.2 ^a,^*	14.76 ± 5.46 ^a,^*
DC	144.9 ± 25.8 ^a,b,#^	53.3 ± 9.7 ^b,^*	13.81 ± 2.37 ^a,^*
NC	239.3 ± 23.7 ^b,†^	56.8 ± 11.2 ^b,^*	6.61 ± 0.58 ^b,#^

Data is expressed as the mean ± SD; “a” indicates the significance of the data that compares group N *vs.* all groups; “b” indicates the significance of the data that compares group D *vs.* groups of crocin(s)-treated rats; *, *p <* 0.05; #, *p <* 0.01; and †, *p <* 0.001.

Diabetes causes an increase in the AGEs and glycated protein formation in the lenses. Table 3 shows the significant differences in the fluorescent AGEs and glycated proteins (*p <* 0.001 and *p <* 0.05, respectively) between the normal and diabetic groups. Administration of crocin(s) had no significant effect on AGE fluorescence and glycated protein of the lenses. Cataract also caused a significant decrease (*p <* 0.05, Table 3) of protein levels in the lens of diabetic rats. Crocin(s) treatment causes a slight increase in both protein and soluble proteins in the lenses of diabetic rats, but these changes were not statistically significant.

**Table 3:** The effect of crocin(s) on various markers of protein glycation and protein content of the rats lenses.

Group Name	Lens AGE (FI%)	Glycated Proteins (μmol HMF/mg Lens prot.)	Total Protein (mg/Lens)	Soluble Protein (mg/Lens)
N	18.0 ± 2.2	0.55 ± 0.01	17.40 ± 0.47	13.72 ± 1.23
D	70.1 ± 5.7 ^a,†^	1.22 ± 0.22 ^a,^*	11.89 ± 1.39 ^a,^*	5.45 ± 1.85 ^a,#^
DC	65.4 ± 7.7 ^a,†^	1.35 ± 0.42 ^a,#^	12.98 ± 2.60 ^a,^*	6.87 ± 1.11^a,#^
NC	19.3 ± 4.6 ^b,†^	0.57 ± 0.17 ^b,^*	17.63 ± 1.15 ^b,#^	14.11 ± 1.32 ^b,#^

Data is expressed as the mean ± SD; “a” indicates the significance of the data that compares group N *vs.* all groups; “b” indicates the significance of the data that compares group D *vs.* groups of crocin(s)-treated rats; *, *p <* 0.05; #, *p <* 0.01; and †, *p <* 0.001.

## 3. Discussion

The aim of the present study was to investigate the role of crocin(s) as an anti-cataractogenic agent. Crocin(s) affects various oxidative markers in the serum and the lenses of the mentioned animals. In addition, crocin(s) inhibited glycation, aggregation and inactivation of α-crystallin.

Diabetes mellitus is one of the reasons of cataract development in animal and human; and it has been shown that the incidence of diabetic cataract rises with increasing prevalence of diabetes. Three major mechanisms of diabetic cataract are: hyperglycemia that causes the formation of glycated proteins in the lens, oxidative stress and increased polyol pathway activity [3]. Different studies indicated that some antioxidants, antiglycating agents, and aldose reductase inhibitors slow down the progression of cataract in diabetic subjects [22,23,24,25,26,27].

In the recent decade, various medicinal properties have been reported for crocin(s) [17,18,28], as the important water soluble apocarotenoids of plant origin [13,29]. It is a powerful antioxidant [14,30,31,32,33] with hypoglycemic [15,33] and hypolipidemic [15,34] activities. Despite to the continuously increasing literature on the various beneficial effects of saffron and its main components, crocin(s), in several different diseases, although the preventive effect of saffron on selenite-induced cataract has been reported [35], but there is no information about the potential of crocin(s) as the anti-cataractogenic agent and its mechanism of action.

It has been previously shown that 2 h after oral administration of crocin(s) in mice, most of it appeared in the feces [36]. Therefore, in the present study, crocin(s) was administered through i.p. injection. Safety evaluation studies also indicated no toxic effect of saffron and crocin(s) at the administered dose in both animal studies and human subject [37,38,39].

The obtained data here indicated the significant reduction of the serum glucose level of type 1 diabetic rats after four weeks of crocin(s) treatment. We also showed the hypoglycemic effect of crocin(s) in type 2 diabetic rats, previously [15]. Garcia-Olmo *et al.* have also reported a slight decrease in the serum glucose level of rats that had suffered from colon cancer and were treated with crocin(s) [40]. We also showed previously that crocin(s) decreased the insulin resistance in diabetic rats [15].

By decreasing the serum level of glucose, it seems that protein glycation should also be decreased. Our previous study indicated a significant decrease in the AGE formation and HbA1c in type 2 diabetes after crocin(s) administration [15], but the *in vivo* study described here did not show this effect and the amount of HbA1c and AGEs remain high in the crocin(s)-treated type 1 diabetic group, which may be due to the experimental period that was lower in this study—two months—in comparison with the previous study where the changes were observed after three months and continued up to six months.

As shown in Figure 7 and Table 1, the lenses of the diabetic group became dark and were completely turbid. However, the turbidity (opacity) of the lens in the diabetic group treated with crocin(s) was less than the untreated group. Crocin(s) had no effect on the lenses of the normal rats. With the progress of cataract, the reduction of total and soluble proteins of the lenses was accompanied by alteration of the protein profile in this tissue. The underlying mechanisms for these changes are oxidative damage of critical sulfhydryl groups of proteins that causes the formation of cross-links between protein thiol groups, and the AGEs formation; as well as the protein leakage due to the increased membrane permeability induced by the osmotic stress [2,41]. As our *in vitro* results indicated, crocin(s) showed an inhibitory effect on protein aggregation and AGEs formation in the presence of high glucose concentrations. Several studies have also shown that non-enzymatic glycation of α-crystallin alters protein stability and leads to protein cross-linking, aggregation, insolubilization, and increased AGE fluorescence, which are associated with the reduction of surface hydrophobicity and chaperone activity of this protein [2,8]. All of the mentioned results were also obtained in the present study under *in vitro* conditions. Such a result was also seen in the rats, but maybe due to the limited treatment time, this effect was not significant.

The CD spectra of α-crystallin in the absence and presence of glucose or crocin(s) were not significantly altered. Since α-crystallin is a very stable protein, the fraction of protein that remained in the solution had no changes in the secondary structure, but the aggregates and cross-linked protein due to glycation was in the insoluble fraction and remained at the upper part of the electrophoresis (SDS PAGE).

Several small molecules are reported to modulate the chaperone activity of α-crystallin under various conditions, including *in vitro* glycation and diabetes [41,42,43]. Similar to crocin(s), most of these molecules exert their effects by inhibiting the non-enzymatic glycation and oxidative damage of α-crystallin and preserving its chaperone activity to prevent cataracts under diabetic conditions.

GSH, is a reducing tripeptide, unusually found at high levels in the lens, that plays an important role in the maintenance of the reduced state of this tissue. GSH’s role is detoxification of potentially damaging oxidants and protection of the thiol groups of proteins against oxidation. It has been shown that the amount of GSH decreases with age and in most types of cataract [24,41]. Furthermore, the alteration of the activities of the antioxidant enzymes such as CAT and SOD has also been reported in different types of cataract, including diabetic cataract. Similar results were obtained in the present study. Table 2 indicates a significant decrease in the GSH and a significant increase in the CAT activity in the lenses of diabetic rats that were compensated by crocin(s) administration. These changes are consistent with the FRAP alterations in the plasma of diabetic rats (Figure 1). Crocin(s) had no significant effect on the mentioned parameters in the normal rats.

Other enzymes involved in the polyol pathway, such as aldose reductase and sorbitol dehydrogenase were also investigated in the lens of rats. The activities of both enzymes were significantly changed due to diabetes induction, but crocin(s) had no significant effect on them in the experimental period in this study (data not shown).

## 4. Experimental Section

### 4.1. Materials

*Crocus sativus* L. stigmas were collected from Ghaenat (Khorasan Province, Iran). Streptozotocin, oxalic acid, 5-hydroxymethylfurfural (5-HMF), 2-thiobarbituric acid (TBA), triethanolamine, NADPH, NADH, Sephacryl S-300 HR, protein and super oxide dismutase (SOD) quantification kits were purchased from Sigma Chemical Company (St. Louis, MO, USA). All other chemicals and solvents were of analytical grade.

Crocin(s) or total crocin, which contains all forms of glycosyl esters of crocetin, was extracted and purified from the dried stigmas of *Crocus sativus* L., as explained previously [44]. The main component of this preparation is α-crocin or crocin-1.

For the purification of α-crystallin calf eyeballs were obtained from a local abattoir, and the lenses were dissected out, homogenized in Tris buffer (50 mM) pH 7.2, EDTA (5 mM), dithiothreitol (DTT, 1 mM), NaN_3_ (0.04% *w*/*v*), PMSF (0.1 mM), and centrifuged at 12,000 rpm for 30 min at 5 °C. The supernatant was fractionated by size-exclusion chromatography on a Sephacryl S300HR column. Protein was eluted as the column buffer (Tris buffer containing NaN_3_, at the above mentioned concentrations, pH 7.5) was passed through at a flow rate 20 mL/h [45]. Fractions containing α-crystallin were freeze-dried and stored at −70 °C until use. The purity of α-crystallin was checked by SDS-PAGE.

### 4.2. Methods

#### 4.2.1. *In Vitro* Studies

*Glycation of α-Crystallin:* A stock solution of α-crystallin was prepared by dissolving it in PBS, pH 7.4. This solution was subsequently diluted with glucose solution made in the same buffer to form duplicate incubation mixtures of 5 mg/mL of protein with 500 mM glucose [8] in the absence and presence of crocin(s) (0.35 mM). After being sterilized by filtration (0.22 µm filters, Millipore, Darmstadt, Germany), the solutions were incubated at 37 °C for 4 weeks in capped vials. Aliquots were collected every week and stored at −80 °C until they were analyzed [46,47].

*Non-tryptophan AGE Fluorescence:* Advanced glycation end products (AGEs) or non-tryptophan fluorescence emission was monitored using 0.15 mg/mL protein in 0.1 M sodium phosphate buffer (pH 7.4), with excitation at 370 nm, and emission recorded between 400–500 nm using a spectrofluorometer (Cary Eclipse, Santa Clara, CA, USA).

*Hydrophobicity of α-Crystallin:* The surface hydrophobicity of α-crystallin was investigated as a function of 8-anilinonaphthalene-1-sulfonic acid (ANS) binding to this protein. α-Crystallin (0.1 mg/mL) was incubated with 50 µM ANS for 30 min at room temperature in the dark, and the fluorescence of protein-bound dye was measured by excitation at 390 nm and measuring the emission between 450 and 550 nm using a spectrofluorometer [8].

*Secondary Structure investigation:* CD spectra were measured on a 810 spectropolarimeter (JASCO, Tokyo, Japan). The samples with a concentration of 0.15 mg/mL α-crystallin were used for secondary structure analysis. All measurements were performed at 25 °C. The spectra have been corrected by subtraction of the background solvent spectrum and smoothed for clarity of display. All the data were converted to give specific ellipticity value based on the sample concentration and expressed in units of mean residue molar ellipticity.

*Chaperone Activity Assay:* Chaperone activity of the native and modified *α*-crystallin was assessed by the method of Ghahghaei *et al.* [45]. In this method, aggregation of the protein was determined by measuring the ability of *α*-crystallin to suppress heat-induced aggregation of catalase (CAT) at 60 °C. Heat-induced CAT aggregation was monitored by protein absorbance changes at 360 nm, as a function of time.

*SDS-PAGE:* The formation of high molecular mass (HMM) aggregates, which is formed as a result of protein cross-linking due to glycation, was monitored using SDS/PAGE (12% gel).

#### 4.2.2. *In Vivo* Studies

*Animals:* The experiments were performed using male Wistar rats (7–8 weeks old and weighing 213 ± 21.5 g) which were housed under a 12-h light and 12-h dark cycle at 20–25 °C room temperature. All animals had unlimited access to food and water. Animal care and protocols were in accordance with and approved by the Institutional Animals Ethics Committee of Tarbiat Modares University and conformed to the ARVO Statement for the Use of Animals in Ophthalmic and Vision Research. After one week of adaptation period, 20 rats (10 rats in each group) were randomly selected for the control or normal rats (groups N and NC, with or without crocin(s) treatment, respectively) and 35 rats were single injected intraperitoneally with streptozotocin (65 mg/kg bodyweight) [25]. The control rats (N for normal) were injected with the vehicle only. Three days after streptozotocin administration, only rats having a blood glucose level >15 mmol/L [25] were considered as diabetic (10 rats in each group) and were included in our experiments. Crocin(s) was injected intraperitoneally to the groups under treatment (half of each of the normal and diabetic groups that were named NC and DC, respectively) with the dose of 100 mg/kg BW after 1 week (time zero in the figures and tables) and repeated four times, at 0, 2, 4, and 6 weeks of the experiment. The experiments were finished in 8 weeks.

*Blood Analysis:* Blood samples were collected from the orbit vein, at the beginning, middle and end of the study. EDTA-treated whole blood samples were preserved for HbA1c measurement and serum samples, prepared by 15-min centrifugation of blood at 5000 g to separate clot and stored at −70 °C for further studies. Serum glucose was measured by an enzymatic colorimetric method (ELITech, SEES, France) using a BT 3500 Autoanalyzer (Biotecnica Instruments, Rome, Italy). Measurements of glycated hemoglobin (HbA1c) in blood samples were performed with the reagent kit and DS5 instrument from Drew Scientific Ltd. (Barrow-in-Furness, Cumbria, UK). Advanced glycation end products (AGEs) determination and ferric reducing antioxidant power (FRAP) assay were examined by the method of Kalousova *et al.* [48] and Benzie and Strain [49], respectively. All other parameters (AR, CAT, and *etc.*) were determined by the methods we have previously reported [25].

*Evaluation of Cataract development:* The progression of cataract was monitored biweekly by a hand held ophthalmoscope equipped with a slit lamp by a specialist in the field of small animal eye, without prior knowledge of affiliation of the animal to an experimental group. Cataract formation was scored essentially according to the classification of Suryanarayana *et al.* [50] and named as follows: clear normal lens (0), peripheral vesicles (I), peripheral vesicles and cortical opacities (II), diffuse central opacities (III), and mature cataract (IV). Cataract maturation was considered when, the red fundus reflex was no longer visible through any part of the lens and the lens appeared dull white to the naked eye.

*Lens Collection and Processing:* At the end of 8 weeks, animals were killed and their eyeballs were removed for biochemical evaluation. Eyeballs were soaked in 0.9% neutral normal saline, and the lenses were dissected by the posterior approach, then placed into pre-weighed Eppendorf tubes and frozen at −70 °C until further analysis. A 10% homogenate was prepared from lens in 50 mM phosphate buffer (pH 7.4). The activity of lens enzymes and soluble protein were measured in the soluble fraction of the lens homogenate (15,000× *g* at 4 °C) whereas lens AGEs and total protein were determined in the total homogenate. Various parameters were determined in the Lens preparations as explained previously [25].

*Statistical Analysis:* Data were expressed as mean ± S.D. of at least three repeats of each *in vitro* experiment and/or the data obtained in the rats in each group. One-way ANOVA was used for testing statistical significance between groups. Statistical analysis of the average of the cataract score of the lens opacity was done using the Mann-Whitney U-test. *p <* 0.05 was considered significant. All the data were analyzed by the SPSS 16.0 statistical package.

## 5. Conclusions

In the present *in vivo* and *in vitro* study, we showed for the first time that crocin(s) from saffron could be effective against glycation of lens proteins (α-crystallin) and diabetic cataract. Our *in vitro* results indicated that crocin(s) has a powerful protective effect against glycation of α-crystallin and preserve the structure and chaperone function of his protein, even in the presence of glucose. This effect is due to its antioxidant and antiglycating action. In the *in vivo* study, we also observed the effectiveness of crocin(s) against diabetic cataract in rat. Crocin(s) as an antioxidant and hypoglycemic agent, decreased protein glycation and prevented the cataract formation, but it seems that its effectiveness will be increased in the long-term administration, a subject that should be investigated in the near future.
